# Epidemiology of East Coast fever (*Theileria parva* infection) in Kenya: past, present and the future

**DOI:** 10.1186/1756-3305-5-194

**Published:** 2012-09-07

**Authors:** John Gachohi, Rob Skilton, Frank Hansen, Priscilla Ngumi, Philip Kitala

**Affiliations:** 1 Kenya Agricultural Research Institute, Trypanosomiasis Research Center, P.O. Box 362–00902, Muguga, Kikuyu, Kenya; 2 International Livestock Research Institute (ILRI), Old Naivasha Rd, P.O. Box 30709–00100, Kabete, Nairobi, Kenya; 3 Biosciences Eastern and Central Africa (BecA) – ILRI Hub, Old Naivasha Rd, P.O. Box 30709–00100, Kabete, Nairobi, Kenya; 4 Kenya Agricultural Research Institute, Veterinary Research Center, P.O. Box 32–00902, Muguga, Kikuyu, Kenya; 5 Department of Public Health, Pharmacology and Toxicology, College of Agriculture and Veterinary Sciences, University of Nairobi, P.O. Box 29053–00625, Nairobi, Kenya

**Keywords:** East Coast fever, *Theileria parva* infection, Epidemiological factors, Kenya

## Abstract

In this article, we review the epidemiology of East Coast fever (ECF), a tick-borne infection of cattle, in Kenya. The major factors associated with epidemiology of ECF include the agro-ecological zone (AEZ), livestock production system (LPS) and both animal breed and age. These factors appear to influence the epidemiology of ECF through structured gradients. We further show that the gradients are dynamically shaped by socio-demographic and environmental processes. For a vector-borne disease whose transmission depends on environmental characteristics that influence vector dynamics, a change in the environment implies a change in the epidemiology of the disease. The review recommends that future ECF epidemiological studies should account for these factors and the dynamic interactions between them. In Kenya, ECF control has previously relied predominantly on tick control using acaricides and chemotherapy while ECF immunization is steadily being disseminated. We highlight the contribution of ECF epidemiology and economics in the design of production system and/or geographical area-specific integrated control strategies based on both the dynamic epidemiological risk of the disease and economic impacts of control strategies. In all production systems (except marginal areas), economic analyses demonstrate that integrated control in which ECF immunization is always an important component, can play an important role in the overall control of the disease. Indeed, Kenya has recently approved ECF immunization in all production systems (except in marginal areas). If the infrastructure of the vaccine production and distribution can be heightened, large ECF endemic areas are expected to be endemically stable and the disease controlled. Finally, the review points the way for future research by identifying scenario analyses as a critical methodology on which to base future investigations on how both dynamic livestock management systems and patterns of land use influence the dynamics and complexity of ECF epidemiology and the implications for control.

## Review

### Introduction

East Coast fever (ECF) is a tick-borne disease (TBD) of cattle whose aetiological agent is a protozoan parasite called *Theileria parva.* The parasite is transmitted cyclopropagatively and transstadially by a three-host tick called *Rhipicephalus appendiculatus,* which have dropped from infected cattle during the preceding stage of the life cycle [1]. In cyclopropagative and transstadial transmission, the *T. parva* parasite multiplies and undergoes cyclical changes within two developmental stages (nymphs and adult) of the vector. The epidemiological implication of this kind of transmission is the amplification of the vector’s competence in parasite transmission and the ability to infect more than one host during the vector’s life cycle. The disease is prevalent across the eastern, central, and southern parts of Africa, and has been reported in 11 countries in the region: Kenya, Uganda, Tanzania, Burundi, Rwanda, Malawi, Mozambique, southern Sudan, Democratic Republic of Congo (DRC), Zambia and Zimbabwe [2]. East Coast fever was also reported in Comoros between 2003 and 2004 for the first time [3]. The latter incident was suggested to result from importation of immunized cattle from Tanzania, which were fed upon by naïve ticks that subsequently transmitted the infection to a susceptible local cattle population [3]. About 28 million cattle in the region are at risk and the disease kills at least 1 million cattle per year. Economic losses are concentrated on small-scale resource-poor households [4].

In Kenya, *T. parva* infection poses a significant threat to the livestock sector in two ways: through the economic impact of the disease from cattle morbidity and mortality and production losses in all production systems, as well as from the costs of the measures taken to control ticks and the disease. The costs of acaricide application, which is the primary means of tick control, was estimated to range between US$6 and US$36 per adult animal in Kenya, Tanzania and Uganda [4]. The disease further prevents the introduction of the ECF-susceptible but more productive exotic breeds of cattle, hampering the development of the livestock sector considerably. This loss is termed “lost potential”.

As a vector-borne disease, the epidemiology of ECF is likely to be largely influenced by varying environmental conditions which in turn influence vector dynamics. The motivation for this review arises from the recognition that global change, associated with human population growth and the consequent changes in land use patterns and urbanization, potentially affect the epidemiology of the disease. This article, therefore, has five aims:

1. It reviews the findings on ECF research from the literature and explores epidemiological factors associated with the occurrence of the disease in the different areas of Kenya.

2. It highlights the contribution of veterinary epidemiology in the design of production-specific and/or geographic area control strategies.

3. It examines the influences of socio-demographic and environmental processes in transforming environments through agricultural intensification and urbanization and their link with ECF epidemiology.

4. It explores anecdotal evidence of changing impacts of ECF, their drivers and likely outcomes.

5. It discusses the methodology of scenario analyses as a way to base future investigations on how socio-ecological dynamics influence change in ECF epidemiology.

We concentrate on Kenya guided by the following reasons: (a) Kenya has contrasting eco-climatic conditions that may influence the socio-economic characteristics and epidemiology of ECF, (b) Kenya is unique in having well characterized dynamic and diverse livestock production systems, and, (c) relatively more studies have been conducted in Kenya compared to other East African countries. In Tanzania and Rwanda, production systems are currently evolving into market-oriented systems similar to the Kenyan situation. Thus, the findings and interpretation in this review are expected to form a basis for understanding the evolving disease epidemiology in the whole region.

### Review methodology

A search of peer-reviewed studies on ECF in Kenya was conducted from comprehensive databases including PubMed, ScienceDirect, Swetswise, and CAB direct. The search was extended to available theses, conference proceedings, project reports etc. Keywords were standardized across the databases to produce comparable searches and these were: East Coast fever, *Theileria parva, Rhipicephalus appendiculatus,* epidemiology, prevalence, incidence, cattle, risk factor, Kenya. References of all relevant articles were also searched to identify articles that could have been missed in the search. The search was conducted for all available years in each database. The keyword search produced several tens of articles published as of September 2011. We screened all the articles then identified the most relevant ones for Kenya.

### ECF and associated epidemiological factors

#### ECF prevalence and incidence by production system and agro-ecological zones (AEZs)

This section deals with the prevalence and incidence of *T. parva* infection across the major cattle production systems and agro-ecological zones (AEZ) in Kenya. Cattle production systems in Kenya cover a wide range from traditional extensive systems to modern intensive systems.

**Figure 1:**
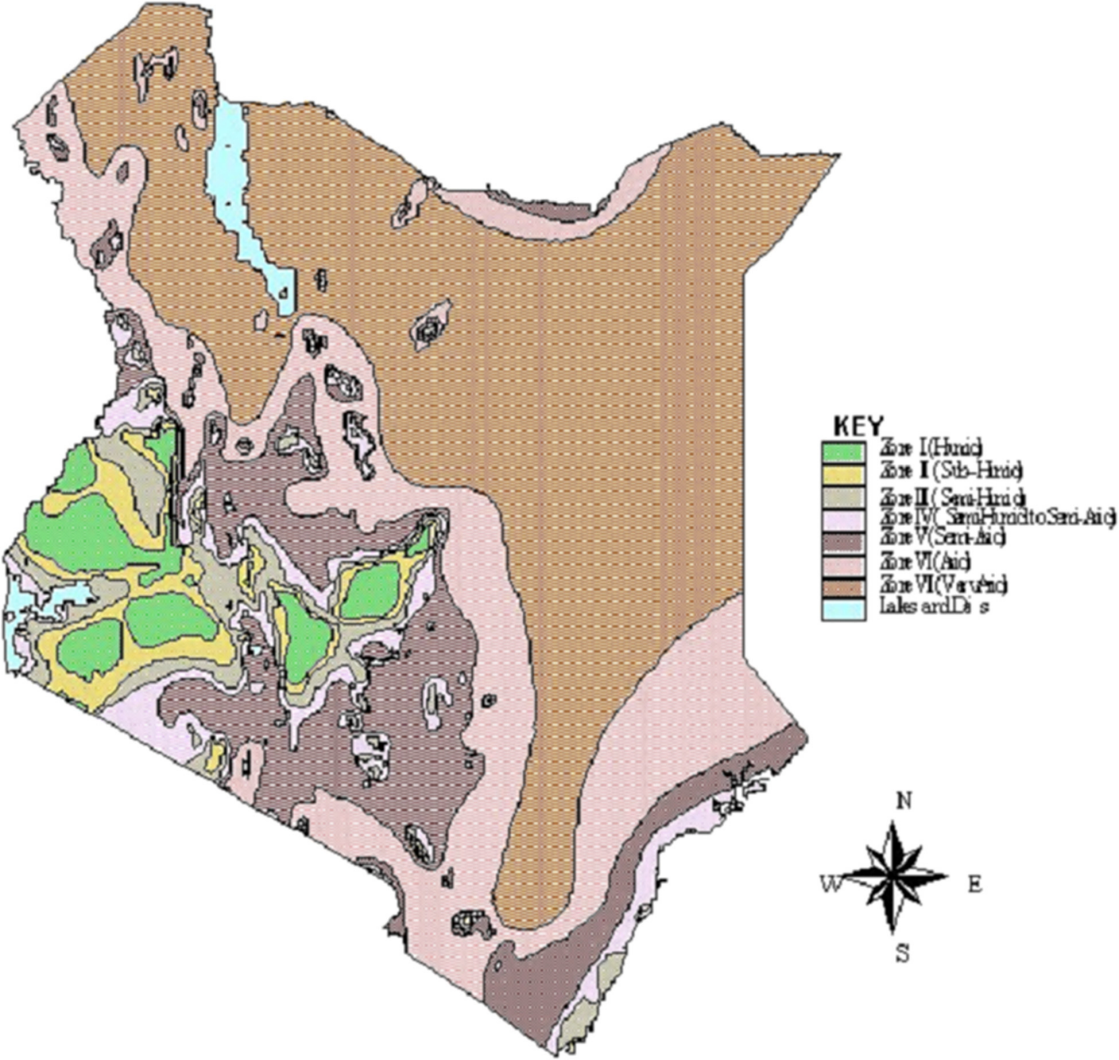
**The major agro-climatic zones in Kenya source: **[5].

Briefly, Kenya is divided into seven agro-climatic zones based on a moisture index [5]. The index represents the annual rainfall expressed as a percentage of potential evaporation. Areas with an index >50% are designated zones I, II and III with potential for substantial vegetation cover. These zones are characterized by smallholder and commercial systems. Areas with an index <50% are designated zones IV, V, VI and VII and these constitute the Kenyan rangelands (Figure 1). Zones IV, V and parts of zone VI are characterized by smallholder mixed systems, commercial ranching and agro-pastoralism. Pure livestock-dependent systems are found in parts of zone VI and VII. The studies that were reviewed are located in each of these zones and were included to achieve standardization across the country. Towards the end of this section, factors influencing the epidemiology of ECF across livestock production systems will be discussed.

#### Traditional extensive systems

Indigenous zebu cattle are kept under traditional extensive management conditions in vast areas of Kenya. These systems are characterized by little or no tick control. Yet, *T. parva* infections in these systems result in little loss in productivity and/or mortality [4]. This phenomenon has been termed ‘endemic stability’ [1]. Endemic stability to ECF has been defined as the state in a cattle population where the large majority (>70%) of the population becomes infected and immune by 6 months of age, and little or no clinical disease occurs [1]. Endemic stability is thought to be the result of complex interactions of several factors such as the high innate resistance of zebu cattle raised in ECF-endemic areas, ability of zebu cattle to rapidly and effectively develop immunity to *T. parva* infection*,* suitable ecological factors for the vector and regular transmission of the parasite in all age groups of cattle population, thus regularly boosting immunity [1]. Consequently, the majority of cattle in such populations are immune. Endemic instability describes a state in which only a small proportion (<30%) of the cattle in the population become infected and immune by 6 months of age leading to a build-up of a susceptible population and, therefore, clinical disease is experienced across all age groups. The latter situation normally exists where animals are kept under low levels of tick challenge. Traditional extensive systems can be divided into traditional crop-livestock and the livestock-dependent systems.

#### Traditional crop-livestock systems

**Figure 2:**
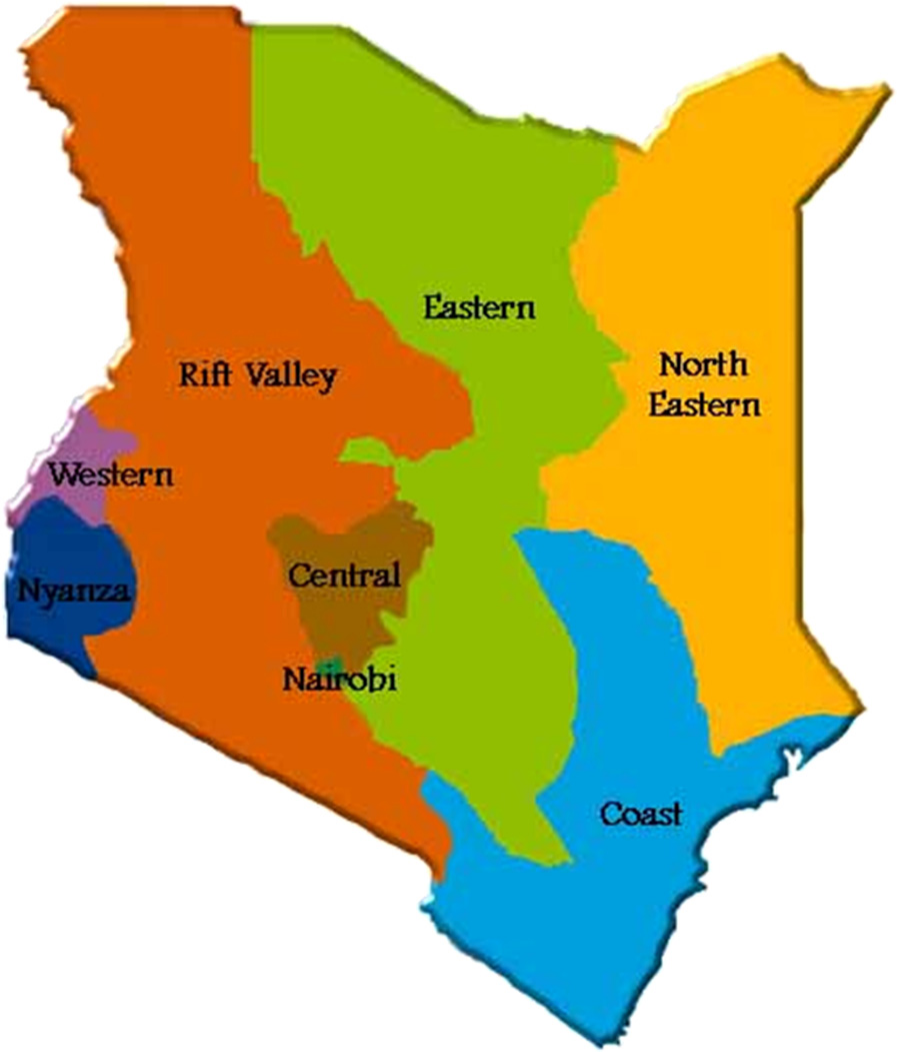
Map of Kenya illustrating Kenya provinces.

Traditional crop-livestock systems integrate indigenous livestock with other subsistence farm enterprises, particularly traditional crop agriculture. The population of cattle in these systems in Kenya was estimated at approximately 5 million [4]. These systems are characterized by variable population immunity to *T. parva,* probably due to the periodic and varying environmental and climatic suitability for the survival and development of the vector. This situation exists in much of the highland areas of Kenya in which zebu cattle are maintained, as well as the Lake Victoria Basin (in parts of both Nyanza and Western provinces) and the Kenyan coastal strip (in Coast Province) (Figure 2) (all mainly in zones III and IV (Figure 1)).

Table 1 shows prevalence and incidence reports and the associated epidemiological factors in traditional crop-livestock systems. Moderate to high antibody prevalence (50 to >70%) and incidence (20 to >50%) levels were reported, accompanied by moderate case-fatality rates (3 to 20%). Variations in prevalence and incidence were assumed to be associated with differences in ecological suitability for the vector across an area, different farm management practices within an area or production system, breed, grazing system, tick control regime and rainfall (Table 1) [6-18].

**Table 1:** ECF prevalence, incidence and case-fatality rates from studies conducted in traditional crop-livestock and livestock-dependent systems in Kenya

**Region/Province**	**District**	**Prevalence (cattle ages sampled)**	**Annual incidence rate**	**Case-fatality rates**	**Epidemiological factors**	**References**
Lake Victoria basin/ Nyanza region	Rusinga island	>70%	NA	NA	Region very suitable for the tick vector	[6]
	Rusinga island	NA	22%	21%		[7]
	Kisumu, Siaya and Bondo	60% (4–18 months)	NA	NA		[8]
Coastal lowlands/ Coast	Kaloleni/Kilifi	22% - 85% (4–18 months)	NA	NA	Region very suitable for the tick vector	[9]
Western Kenya highlands	Uasin Gishu	60%^a^, 73%^b^	32%^a^, 39%^b^	NA	Farm management practices influenced epidemiology	[10]
Central highlands (Central Kenya)	Murang’a (AEZ: UM4*)	72% (6–18 months)	90%	16%	AEZ suitability for tick vector differs, age, breed, grazing system	[11,12]
Western province	Busia district	7% - 8%^c^	NA	NA	-	[13]
Southern Rift Valley (Maasailand)	Trans Mara	~ 100% <6 months	NA	3%	Age	[14,15]
Eastern Province (Arid-semi arid region)	Mbeere District	All age categories 4% – 48%	NA	NA	AEZ suitability for tick vector differs, presence of vector tick on the farm, calf tick control frequency, herd size	[16]
	Machakos District	All age categories 60%	NA	NA	-	[17]
Southern Rift Valley (Maasailand)	Kenya-Tanzania border	NA	NA	30% to 60%	Precipitation levels	[18]

a: Rural area; b: Peri-urban; * Upper midlands 4; c: parasitological data.

#### Livestock-dependent systems

In livestock-dependent systems, indigenous livestock are kept communally and are extensively grazed [4]. These systems include nomadic and transhumant pastoralism and some agro-pastoralism, all of which are found in arid and semi-arid areas of Kenya (mainly from zone V upwards). The population of cattle in pastoral systems in Kenya was estimated at over 4 million [4]. East Coast fever has been identified as the major cause of calf deaths among indigenous cattle in these systems, with mortality rates of 40–80% in unvaccinated calves in Maasai pastoralist herds at the Kenya-Tanzanian border [18,19]. The Maasai pastoralists from the Kajiado District in the south-eastern Rift Valley Province (Figure 2) perceive ECF to be the most important cattle disease [20]. Among the pastoralists, mobility has been a key feature in traditional livestock disease management techniques. However, the ability to move between alternative seasonal pastures is becoming increasingly limited [18]. For many infections, (including TBDs) this mobility used to allow gradual exposure to infections, which stimulated immunity but avoiding serious disease challenge [21].

These systems are, therefore, also characterized by variable levels of population immunity to *T. parva*. However, in certain regions, the pastoralists’ traditional grazing areas lay on very suitable habitats for the tick vector leading to the successful establishment of endemic stability. An example of this scenario has been studied and documented in the Trans-Mara District in the south-western area of the Rift Valley Province (Figure 2): in one study, all calves (n = 116) became infected by 6 months of age with very low ECF-specific mortality (3% up to 6 months of age) [14]. In another study, none of the calves (n = 31) died up to 6 months of age although all developed *Theileria* infections [15]. At the time these studies were carried out (early 80s), this area could as well be characterized as a livestock-wildlife interface with the presence of buffalos among other wildlife species. Although it is not yet clearly evident that buffalos play a big role in the epidemiology of cattle *T. parva* strains [2], transmission of *T. parva* parasites between cattle, buffalo and the ticks cannot be ruled out. This review could not find any observational study that had been conducted in a well-defined livestock-wildlife interface in Kenya.

#### Intensive systems

Intensive systems can be grouped into the commercial and intensive or semi-intensive smallholder dairy systems.

#### Commercial systems

Commercial systems consist of dairy or beef production units in which highly productive exotic breeds of cattle are kept. In the context of ECF, the systems are characterized by intensive acaricide application that leads to the disruption of *T. parva* transmission. This system has become less important in Kenya because the majority of the large farms has collapsed or has been subdivided for human settlement. We could not find any observational study conducted in this system in Kenya from the literature.

#### Intensive/semi-intensive smallholder dairy systems

In Kenya, intensive/semi-intensive smallholder systems play a significant role as they produce >80% of the milk sold in the country [22]. The population of cattle in smallholder dairy systems in Kenya was estimated at approximately 2 million [4]. Generally, these systems are characterized by different management practices at the farm level, agro-ecological characteristics and grazing systems [11]. Consequently, they exhibit varying ECF prevalence, incidence and ECF-specific morbidity and mortality rates.

Table 2 shows the range of antibody prevalence and incidence of ECF in areas where these systems predominate [11,12][23-27]. In Murang’a District in Central Province (Figure 2), the *T. parva* antibody prevalence and incidence were high in the lower agro-ecological zones under open grazing systems. On the other hand, the *T. parva* antibody prevalence and incidence were lower in the higher agro-ecological zones under zero grazing systems (stall-feeding). In the neighboring densely populated Kiambu District in the same province (Figure 2) where exotic breeds predominate, contrasting results to those reported from Murang’a District were found - a generally low ECF-specific mortality risk but moderate to high antibody prevalence to *T. parva* (Table 2) which may be indicators of near endemic stability in the area [23]. This is contrary to earlier assumptions that exotic cattle breeds are associated with endemic instability [1].

**Table 2:** East coast fever prevalence, incidence and case-fatality rates from studies conducted in intensive/semi-intensive smallholder dairy systems in Kenya

**Region**	**District /area**	**Prevalence (cattle ages sampled)**	**Annual incidence rates**	**Case-fatality rates**	**Epidemiological factors**	**References**
Central highlands	Kiambu	41%-55%			Age	[23]
	Murang’a	18%^a^, 72%^b^ (6–18 months)	54%^c^ 74%^d^ 86%^e^, 110%^f^	6%^c^, 5%^d^ 9%^e^, 16%^f^	AEZ suitability for tick vector, age, breed, grazing system	[11,12]
Coastal lowlands	Kaloleni/ Kilifi	57%^g^, 79%^h^ (adult)			Age, AEZ, grazing system	[24]
	Kaloleni/ Kilifi	18%^g^ 48%^h^ (<18 months)	6.0%^g^ - 50.4%^g^, 10.8%^h^ - 87.6%^h^	13%^g^, 31%^h^	Age, AEZ, grazing system	[25]
	Kwale		23%*	11%*	Age, grazing system	[26]
Central Rift Valley	Nakuru		22%^j^, 33%^k^		Grazing system	[27]

a: higher elevation AEZ; b: lower elevation AEZ; c: zero grazing/higher AEZ elevation stratum; d: zero grazing stratum/lower AEZ elevation stratum; e: free grazing/higher AEZ elevation stratum; f: free grazing/ lower AEZ elevation stratum; g: zero grazing; h: free grazing; j: semi-zero grazing; k: free grazing; *parasitological data.

A longitudinal study in Nakuru District located in the central Rift Valley Province (Figure 2) within a single ecological zone, reported comparable results to the above-described studies in cattle under differing production systems [27] (Table 2). In that study, there was no significant difference in the prevalence between the adult and the young stock in the three production systems studied. Generally, however, there was higher *T. parva* infection antibody prevalence in open (unrestricted) grazing and semi-zero grazing systems compared to a zero grazing (stall feeding) system.

In contrast to the studies in the Central Province, two other studies [24,25] concentrated on two major production systems (zero grazing and open grazing) within two AEZs (coconut-cassava and cashew nut-cassava zones) at the Kenyan Coast Province (Figure 2). East Coast fever was the predominant disease diagnosed at the coast and accounted for over two-thirds of all reported deaths. In the cross-sectional study, open grazed cattle in both AEZs had a mean seroprevalence of >75% for both dairy and zebu breeds although seroprevalences in calves <6 months of age in the cashew nut-cassava zone were less than 50% [24]. This clearly indicated endemic stability in open grazing systems in both zones regardless of cattle breed. In the longitudinal study in the same area, the mean monthly ECF incidence rate in animals’ ≤18 months of age was lower in zero-grazing compared to the open grazing systems [25]. Overall, the ECF prevalence and incidence in this region was higher than those reported from all other areas in Kenya (Table 2). Whereas there was no association between age and antibody prevalence in the cross-sectional study [24], increasing age was a significant factor in determining *T. parva* antibody prevalence in the longitudinal study [25]. In the open grazing system, a gradual increase in antibody prevalence with age to over 90% in cattle greater than 18 months of age was found [25]. Surprisingly, although more herds under the open grazing system than in the zero grazing system were affected by *T. parva* infections, the difference between the two grazing systems was not statistically significant. Similarly, the differences in ECF incidence risk and ECF case-fatality between the two grazing systems was not significant.

**Figure 3:**
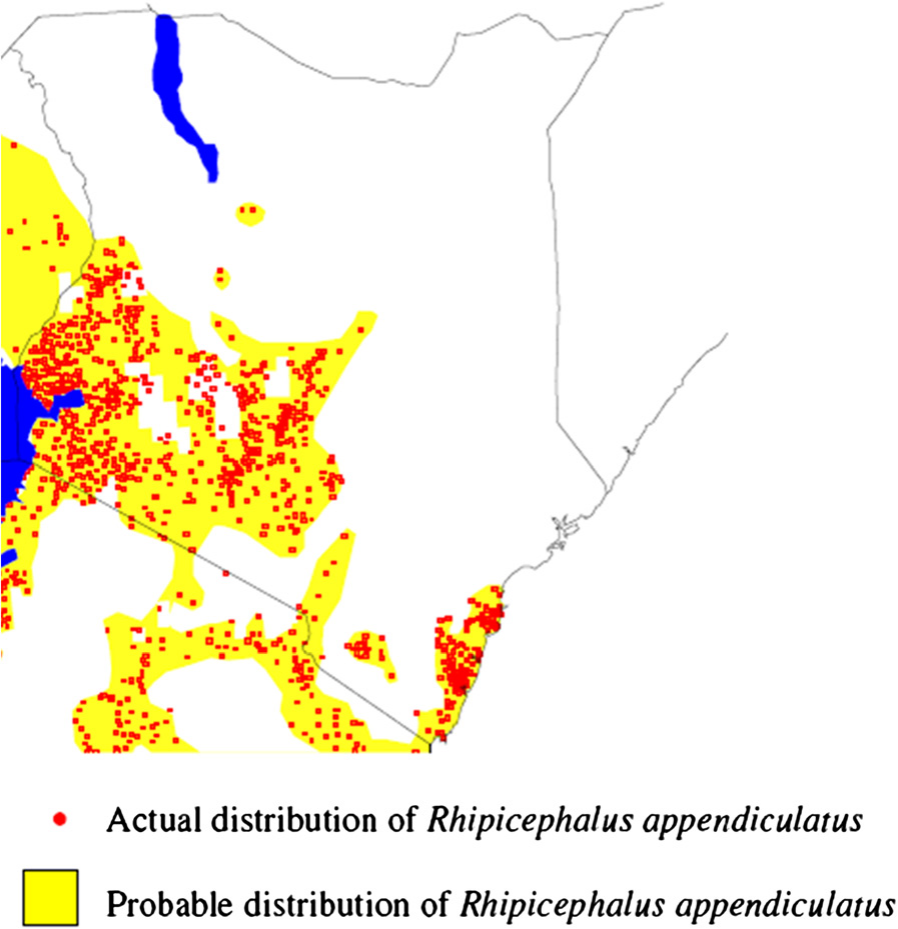
**Map of Kenya illustrating the distribution of ECF vector tick. Source: **[28].

Geographically, Figure 3 illustrates the actual and probable distribution of *Rhipicephalus appendiculatus* in Kenya. This may correspond to the distribution of *T. parva* as well.

### Epidemiological factors associated with prevalence and incidence of East Coast fever

**Figure 4:**
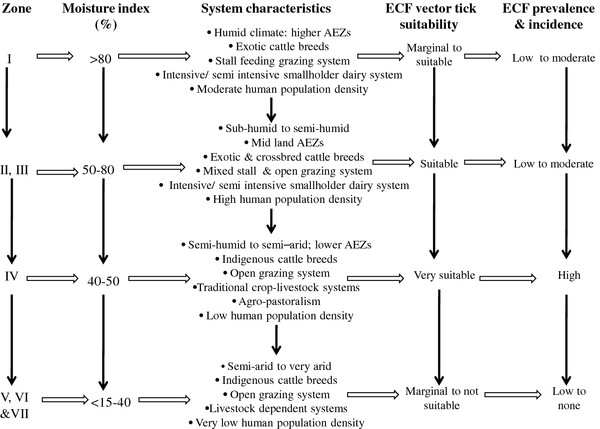
Illustration of gradient of effects of AEZs, farming systems, ECF vector suitability characteristics and corresponding ECF qualitative prevalence and incidence levels in Kenya.

A closer look at the epidemiological factors affecting the prevalence and incidence of ECF reveals a gradient of effects on the epidemiology of ECF (Figure 4).

#### The agro-ecological zone (AEZ) gradient

**Figure 5:**
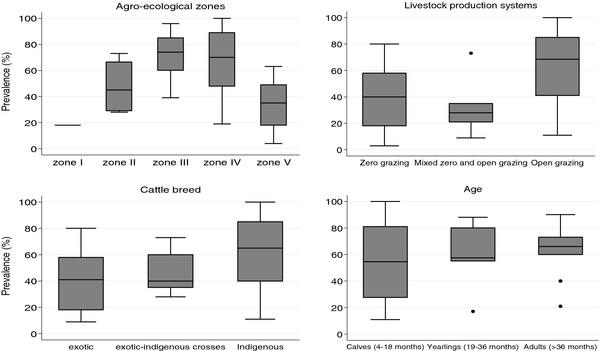
**Box and whisker plots illustrating the distribution of prevalence (%) values by the identified factors obtained from the reviewed studies.** The upper whisker represents the maximum value whereas the lower whisker represents the minimum value. The band at the middle of the box is the median value. The circles outside the whiskers are the ‘far out’ values. Prevalence median values progressively increase by AEZs up to zone IV then subside in zone V. Prevalence median values for open grazing system and for indigenous cattle are higher than for other production systems and cattle breeds respectively. Prevalence median values progressively increase with age.

When all prevalence values from the reviewed studies were examined by AEZs, the median values progressively increased from zone I up to zone III then subsided in subsequent zones (Figure 5). The AEZ gradient is largely determined by the variation in climatic suitability for the tick vector moving from tick suitable areas through marginal areas to areas where the tick cannot survive (Figure 4). Consequently, the gradient gives rise to the different ECF epidemiological states [1]. Endemic stability is expected to occur in tick suitable areas. Endemic instability and epidemic ECF occur in marginal areas and regions where the tick can barely survive.

*Rhipicephalus appendiculatus* in Kenya is found from sea level to over 8,000 feet in areas where there is annual rainfall of over 500 mm [1] (zone II to VI). Areas that are more suitable for ticks are warmer and more humid with landscapes characterized by a mixture of grass and tree cover (savannah woodland) [1]. These are found in the Lake Victoria basin (in Nyanza and parts of Western provinces), the Kenyan coastal region and some parts of the central and eastern highlands, representing zones II, III and IV (Figures 1,2 and 3). In these areas, antibody prevalence is high (Figure 4). Indeed, after ECF was first reported in Kenya in 1904, reports indicate that the disease spread fast from some of these foci (Lake Victoria basin and the Kenyan coastal region) [1]. In these initial foci areas, low mortality rates were found in the absence of tick control [7] indicating endemic stability. At the coast, a seasonal-independent, all-year round occurrence of *T. parva* infections was reported [25]. However, even within a generally suitable region, differences in the suitability of habitats have been suggested based on vector tick counts on animals and *T. parva* prevalence and incidence [9,11,16]. Following on the AEZ gradient, vector unsuitable areas are harsh, hot and dry, have sparse vegetation and open grasslands. In the context of ECF, these conditions can be found particularly in semi-arid North Eastern [29] and the upper parts of Eastern provinces in Kenya (Figures 2 and 3), which represent zones V to VII (Figure 1). In these areas prevalence is low (Figure 4). Unsuitable areas also include regions where overgrazing and environmental degradation has occurred and in deep forests [1,2].

#### The livestock production systems gradient

The livestock production system has an important influence as far as the exposure of cattle to the different ecological characteristics is concerned. In Kenya, these range from unrestricted tick exposure in open grazing systems through limited exposure in mixed grazing, where animals are alternately open grazed and stall fed, to no exposure where cattle are kept under confinement as in the smallholder zero-grazing units. These differential exposures result in considerable differences in tick infestation levels and corresponding infection prevalence and incidence [11,12,24,25] (Figure 5). As ECF can only be transmitted by ticks that have dropped from infected cattle during the preceding stage of the life cycle, the spatial spread of infection is mainly through cattle movement during grazing [30]. In certain circumstances, however, open grazed cattle could be the source of tick-borne infections in both ticks and zero-grazed cattle either in the same farm or in the neighborhood if grazing areas are also the main source of cut-and-carried forage for the zero-grazed cattle [24,25].

The grazing system employed in an area tends to correspond to the consequences of social, economic, technological and demographic processes. Thus, zero grazing systems, characterized by exotic cattle breeds is mainly practiced in the medium- to high-potential farming areas (zones I to III) in response to decreasing land sizes due to increasing human population and consequently higher livestock product demand [31] (Figure 4). Only a few cattle can be supported in a zero grazing system, as the available land is small. By default, these cattle ought to be highly productive and ECF-susceptible exotic breeds for income generation and satisfaction of the ready milk markets. In Murang’a District in Central Province (Figure 2), for example, the smallholder dairy system predominates in the upper highland and midland AEZs [11,12] where the human population density is >576 people/km^2^[32]. Lower AEZs are characterized by lower human densities (<380 people/km^2^) [32], crossbred and indigenous cattle breeds and open grazing systems (Figure 4). This situation is likely to be similar in other districts.

#### The genetic gradient

The host genetic gradient was examined experimentally [33] and reflects the variation in susceptibility of cattle to *T. parva* infection. In the studies reviewed, this gradient was somewhat obscure between the exotic animals and the crosses between the indigenous and exotic cattle (Figure 5). The median prevalence for indigenous cattle was, however, larger than for the other breeds (Figure 5). Purebred taurine cattle bred under tick-free conditions are highly susceptible to ECF. Zebu breeds (such as improved and unimproved Boran) bred in tick-free conditions are moderately susceptible to the disease. However, zebu cattle bred in ECF endemic areas have low susceptibility for the disease. As already indicated, this factor is partly associated with grazing systems as the ECF susceptible exotic breeds are mainly kept in smallholder dairy systems under restricted tick exposure whereas the ECF resistant indigenous breeds are kept under an open grazing system that permits exposure to infected ticks (Figure 4).

#### The age gradient

Increasing age is associated with increased *T. parva* seroprevalence (Figure 5) and this was particularly noted in studies conducted at the Kenyan coast. This result may be expected since age is a proxy for exposure time and, as antibodies for *T. parva* persist in the circulation for as long as six months, the seroprevalence in a population is likely to increase with age [34].

### The role of epidemiology in the control of ECF

It is beyond the scope of this paper to discuss the details of ECF control. However, we will briefly describe the main control methods and then highlight their applications in the context of ECF epidemiology. The main methods in the control of ECF include tick control, host immunization and chemotherapy and integrated control that combines any of the methods. Tick control methods include direct application of acaricides to cattle through dipping, spray races, hand spray, pour-ons, and hand dressing. However, acaricides have their own disadvantages: they are expensive, ticks can easily develop resistance to them and they can be detrimental to the environment (reviewed by [35]).

The ECF immunization concept arose from observations of naturally acquired immunity and involves an elaborate infection-and-treatment strategy (ITM). The live *T. parva* parasites are inoculated into an animal while simultaneously treating the animal with a long-acting tetracycline antibiotic. This procedure results in a mild and controlled reaction to the parasite infection that leads to development of immunity to subsequent infections [19]. The immunity lasts up to three years in the absence of further tick infestations but is life-long if infected ticks continue to challenge the immunised animal regularly [19].

In the chemotherapy of ECF, tetracycline antibiotic was probably the first compound to be used in 1953. As tetracycline therapeutic effects were limited to only the early stages of the disease, more effective derivatives of naphthoquinone compound (parvaquone and buparvaquone) were discovered in the late 1970s. These drugs are, however, expensive and this reduces their use in the field [2].

The preceding discussion on ECF epidemiology illustrates that the differences in epidemiology of ECF in an area/production system is a major determinant of the choice of methods for its control. Livestock-dependent and certain forms of traditional crop-livestock production systems are characterized by little use of acaricides and therapeutic resources leading to what can be termed as “natural” disease control [1]. Increased exploitation of the livestock genetic resistance is likely to remain the most cost-effective strategy in these systems. However, because of the high mortality in young non-immune calves [18,19], integration of the control methods by combining immunization and seasonal tick control could be more beneficial. In Kenya, a national roll-out of the ECF vaccine (Muguga cocktail [36]) was approved for use on ECF-endemic livestock dependent production systems in the Maasai ecosystem in 2005 [37]. The districts where the vaccine had been approved included Narok, Kajiado and Trans Mara districts in southern Rift Valley Province (Figure 2). ITM adoption in livestock-dependent systems neighbouring these districts in northern Tanzania is steadily increasing [19]. Moreover, a study conducted in northern Tanzania to assess the impact of the control methods showed that ITM was the most cost-effective control option particularly when compared with treatment of cases [38]. Similar results were reported in a study on financial analysis of ECF control strategies in traditionally managed Sanga cattle in Zambia - a higher net present value for integrated immunisation and seasonal tick-control strategies [39]. The higher levels of adoption in the Maasai pastoral system has probably been boosted by production of a vaccine of much higher quality, use of a higher dose of antibiotics during ITM and development of molecular tools to support field delivery of the vaccine [40].

In endemic unstable areas, which are characterized by a wider range of intermediate antibody prevalence levels, control measures, for instance, immunization, depend on the proportion of the susceptible population [9]. The goal of such an approach is to increase the proportion of immune animals to endemic stability status and minimize tick control to allow the infected ticks to naturally sustain endemic stability through continuous challenge. Indeed, a study on economic impacts of cattle ECF immunization in a traditional crop-livestock system in Kilifi District at the Kenyan coast, demonstrated that ECF ITM integrated control strategies were financially and economically more profitable than acaricides-based strategies [41].

In the smallholder dairy production systems characterized by intensive and semi-intensive management conditions, the control of ECF is variable and dynamic due to differences in farmer risk averseness and perceptions of decreased disease incidences associated with low tick exposure [11]. The study by Nyangito *et al*. 1996 [41] found that ECF immunization strategies are financially and economically viable on smallholder dairy production systems in Uasin Gishu District in Central Rift Valley Province (Figure 2). The issue of safety while using the live vaccine in exotic dairy cattle that had hindered immunization roll-out in smallholder dairy production systems was recently resolved when a vaccination trial involving more than 3000 exotic dairy cattle in different ECF endemic areas of Kenya reported that the vaccine is safe and efficacious [37]. Subsequently, the roll-out was extended to all dairy ECF-endemic production systems and areas [37].

In *T. parva* marginal and clean areas, effective population immunity is very low or non-existent. This is mainly because of the periodic unsuitability of the climate for the development and survival of the tick [1]. It is logistically and financially difficult to sustain immunization of very large proportions of susceptible livestock in such areas and, moreover, there would be little or no challenge from naturally infected ticks to sustain the immunity. Indeed, the recent extension of the National roll-out [37] excluded certain areas in the North-Eastern and upper Eastern provinces and Rift Valley provinces (Figures 2 and 3) which are free of the disease. In such areas, the most cost-effective control method seems to be strategic tick control during periods when the vector is found and early and effective treatment in cases of successful transmission.

### A framework for understanding and interpreting ECF epidemiology in Kenya

#### The influence of socio-demographic and environmental processes

Characteristics of a particular livestock production system emerge from interactions of situational micro-level environmental behaviours of social, economic and demographic processes such as increased human population, agricultural development, land use changes and urbanization. Population growth is the most likely driver of land use and environmental change particularly in higher AEZs [42]. An illustrative example is the situation in the Kenyan central highlands. Due to increased population pressure, land is divided between generations [32,42]. To improve productivity of their small plots, the smallholder farmers turn the formerly tick infested pastures into farmland where they plant improved fodder which cannot be infested by ticks. The fodder is used to feed the zero-grazed cattle. Socio-demographic and environmental processes have, therefore, led to the evolution of a new production system (smallholder dairy) whose characteristics not only minimizes exposure of cattle to infected ticks, but also greatly perturbs the tick habitats [2,11,12]. The “artificially” created environments that are less suitable for ticks alter the local epidemiology of the disease. Growth of smallholder dairy systems in these areas is, therefore, likely to be associated with reduced incidence of ECF.

The situation becomes increasingly complex when one closely examines variations within systems from different areas or regions, for example, the differences in prevalence between zero grazing systems in Kiambu and Murang’a districts in Central Province and in Coast Province. Kiambu District borders the capital city of Nairobi. The proximity of the city, with its established milk markets and the availability of modern dairy technology means that in Kiambu, dairy systems are characterized by higher levels of management and more intensive input of labour and capital. Thus, ECF cases are likely to be managed successfully with minimal fatalities in Kiambu. This may explain why the detected seroprevalence was higher, yet this was associated with exotic cattle [34]. This is an example of how the epidemiology of ECF may be altered by effective management of the disease that is linked to improved veterinary services delivery and high levels of farmer awareness which are in turn associated with urbanization.

In areas where the rate of dairy intensification is lower, the picture is different. At the coast, the smallholder dairy farmers usually keep the local zebu breeds [9,24,25]. Open grazed zebu cattle are mainly *T. parva* carriers and may serve as a potential source of infected ticks to zero-grazed cattle through cut-and-carry fodder from contaminated pastures. This observation may explain the higher ECF prevalence, incidence and case-fatality rates at the coast [24,25]. It is expected that similar conditions exist in farms and regions with traditional smallholder mixed crop-livestock systems where farmers are in the process of intensifying dairy production, like the medium and lower zones in Murang’a District. The difference in the ECF epidemiology at the coast could be further related to the AEZ gradient: the coastal strip of eastern Africa is regarded as one of the areas with very favorable climatic conditions for *R. appendiculatus*[43].

### Use of scenario analysis to understand the epidemiology of ECF

Understanding the consequences of the social, economic and demographic processes in relation to the changing ECF epidemiology requires the generation of empirical knowledge in three key areas by:

1. Studies to estimate the risks of ECF and to forecast the potential impacts of comparable exposures both in different geographical regions and in time;

2. Studies to investigate early evidence of changes in epidemiological risk occurring in production systems in response to social, economic and demographic processes and;

3. Using existing knowledge to develop models of future epidemiological outcomes in relation to defined social, economic and demographic process scenarios.

From the preceding discussion, an emerging hypothesis is that dairy intensification is correlated with decreased incidence and impacts of ECF. However, it is hard to predict the rate of intensification in any system as individual farmers differ in the extent to which they cope with the trends of social, economic and demographic processes. Secondly, veterinary monitoring and surveillance systems in developing countries are currently unable to provide data on disease occurrence that are adequately standardized and reliable enough to allow comparisons over long time periods or between locations [44]. Preliminary findings in central Kenya [11,12] linked the social, economic and demographic processes with perturbations in the ecological equilibrium between hosts, vectors and parasites leading to changes in the frequency and distribution of ECF.

If more data becomes available, it will become easier to evaluate the change in ECF risks posed to the cattle population as a result of changes in the social, economic and demographic processes. The effects of these processes cannot, however, be investigated in experimental studies; the consequences of the future effects can, however, be quantified by scenario analysis. Scenarios are plausible and often simplified descriptions of possible ways of how certain parameters may change in the future, based on a coherent and internally consistent set of assumptions about key driving forces and relationships [45]. Scenarios are neither specific predictions nor forecasts, but they provide a starting point for investigating questions about an uncertain future and for visualizing alternative futures [45]. In a disease context, the use of scenario analysis may, in addition, help to explore potential response strategies.

One way to undertake scenario analysis is to develop, test and validate scenario-based predictive models. Ideally, such models may include scenarios of future societal, economic and technological conditions. For example, it is possible to construct a simulation model based on conditions and processes similar to those experienced in the recent past in specific production systems in an integrated manner. As the highlighted epidemiological factors may interact in complex ways to determine disease risk, scenario-based predictive modelling is more useful as it diverges from the current “risk factor” approach to epidemiology, which emphasizes on independent effect measures for exposures. Secondly, scenario-based predictive modelling can account for nonlinearity and feedbacks. Nonlinearity describes a situation where the change in disease risk is not linearly associated with change in exposure. Feedback describes a situation where disease can modulate exposure just as exposure can modulate disease. The preceding discussion has an excellent example: dynamic changes in AEZ features can predict dynamic changes in livestock production system features in an area. On the other hand, livestock production system features can predict AEZ. These two variables thus support each other in a positive feedback loop with implications on ECF risk. These relationships are likely to exhibit a nonlinear behaviour because of augmentation at each turn of the iteration in a model (or in an ideal practical manner). Thus, these models should be based on the physical and biological understanding and dynamism of the production systems. Scenario-based predictive modelling can be integrated with infection dynamic and landscape models to contribute in three critical aspects in ECF epidemiology and, consequently enhance decision making:

1. To better describe and predict the dynamic relationship between varying antibody prevalence, ECF incidence, case-fatality rates and other important factors associated with the wide range of intermediate antibody levels in endemic unstable areas.

2. To capture the complexity associated with current disease distribution dynamics and the effect of projected changes in social, economic and demographic processes on dynamics of economic impacts and control.

3. To simulate (cost) effectiveness of single and combined control strategies under predicted levels of disease risk in specific production systems.

Scenario analysis has, however, its own limitations. By focusing on the past and possible future conditions and assumptions in their development, scenarios are highly subjective and this may lead to bias and further scenario uncertainty. Lack of essential information involving data and parameters and projections in system behaviour can lead to erroneous assumptions. Erroneous assumptions, in turn, limit fuller understanding concerning scenarios and their incorporation and application. If scenarios are used in a probabilistic manner, then the scenario becomes restricted in its application. In addition, if an alternative future is determined by a set of conditions that cannot be currently estimated, then no probability can be assigned to such an uncertain outcome. Lastly, lack of political plausibility in scenario development can damage its credibility, particularly if scenarios are generated by experts solely from scientific knowledge. As such, stakeholder-derived scenarios usually have greater political plausibility and public recognition than expert-driven scenarios [46].

### Integrating the livestock keepers perspectives in the changing impact analyses

In understanding the social, economic and demographic processes and their effects, it is important to incorporate the livestock keepers’ perspectives and decisions. The study by Gitau *et al.*[11] in Central Province reported minimal and irregular use of acaricides in zero-grazing farms, which was an indication that with increasing intensification of dairy production, the use of acaricides may decline. Further, Gachohi *et al*. [16] reported differential tick control practices that corresponded with changing *T. parva* infection risk. The differential use of acaricides probably reflected a number of factors, key among them being (i) the changing farmers' perception of decreased ECF-risk associated with low tick exposure (ii) the capital-poor intensification strategy and (iii) previous experience of ECF in terms of mortality and the high cost of treatment [11]. This illustrates an important form of adaptive response by livestock keepers to the shifting disease risks that exploits beneficial opportunities provided by changes in the social, economic and demographic processes.

### Effective detection and measurement of social, economic and demographic effects through interdisciplinary collaboration

To provide secure evidence of the impact of social, economic and demographic processes on the changing epidemiology of ECF, the existing veterinary monitoring and surveillance systems need to be strengthened. The future challenge is the continuous need to describe and quantify the changing risk and distribution of ECF. An accurate monitoring of ECF in the present situation would also provide a baseline for epidemiological studies and predictive models. Detection and measurement of social, economic and demographic processes and any changes in tick habitats should be effected through interdisciplinary collaboration between socio-economists, ecologists and veterinary epidemiologists in order to expand the breadth of information. Moreover, considering that livestock keeper behaviour has not been previously integrated in impact analyses, agricultural economists need to collaborate with veterinary epidemiologists in highlighting behaviour that would lead to a better understanding of the changing impacts in consequence to socio-economic changes.

## Conclusions

This review has found that all epidemiological states of ECF are evident in Kenya based on geographical region and/or livestock production systems. At any particular moment (the present), the epidemiological states of ECF are a result of interaction of broad environment and animal genetic factors. Interestingly, these factors influence the epidemiology and the control of ECF through structured gradients. In all production systems (except marginal areas), economic analyses demonstrate the value of integrated control in which ECF immunization is always a necessary component. To illustrate the critical role of immunization, Kenya has recently approved ECF immunization in all production systems (except in marginal areas). This is probably due to the resultant lowered calf mortality and higher weight gains and the natural boosting of the immunity by infected ticks. If ECF immunization can be sustained, large areas are expected to be endemically stable. However, the effects of the gradients are not static as they are part of the dynamic socio-demographic and environmental processes and, therefore, the ‘future’ will be different from the ‘present’. An example was illustrated in the emerging smallholder market-oriented production system that perturbs the ecological habitats of ticks and thereby altering the frequency and distribution of the disease and consequently affecting the epidemiology of the disease. However, it is difficult to predict the rate of development of a “new” system as individual farmers differ in the extent to which they cope with the trends of social, economic and demographic processes and this also depends on the magnitude and rate of change of key factors driving human populations and the demand for livestock products. Scenario analysis is a critical area for such research because it can identify a range of plausible consequences of the changing environmental processes under conditions of uncertainty. The review recommends interdisciplinary collaboration between veterinary epidemiologists, socio- and agricultural economists and ecologists not only to expand the breadth of information but also in understanding the dynamism and complexity of the whole process.

## Competing interests

The author(s) declare that they have no competing interests.

## Authors’ contributions

Conceived the idea: JG, RS; Article design and structure: JG, FH; Literature search: JG; Wrote the article: JG; Interpretation and discussion of findings: JG, RS, FH, PN, PK; Critical revision: RS, FH, PN, and PK. All authors read and approved the final version of the manuscript.
